# Supplementary material for: A functional genetic toolbox for human tissue-derived organoids
Source: eLife. 2021 Oct 6;10:e67886. doi: 10.7554/eLife.67886 (PMC8553336; doi:10.7554/eLife.67886)
Supplement: Source data 1. [file elife-67886-supp4.zip › Summary of gel and blots source data after adjusted.pdf]

Figure 3e

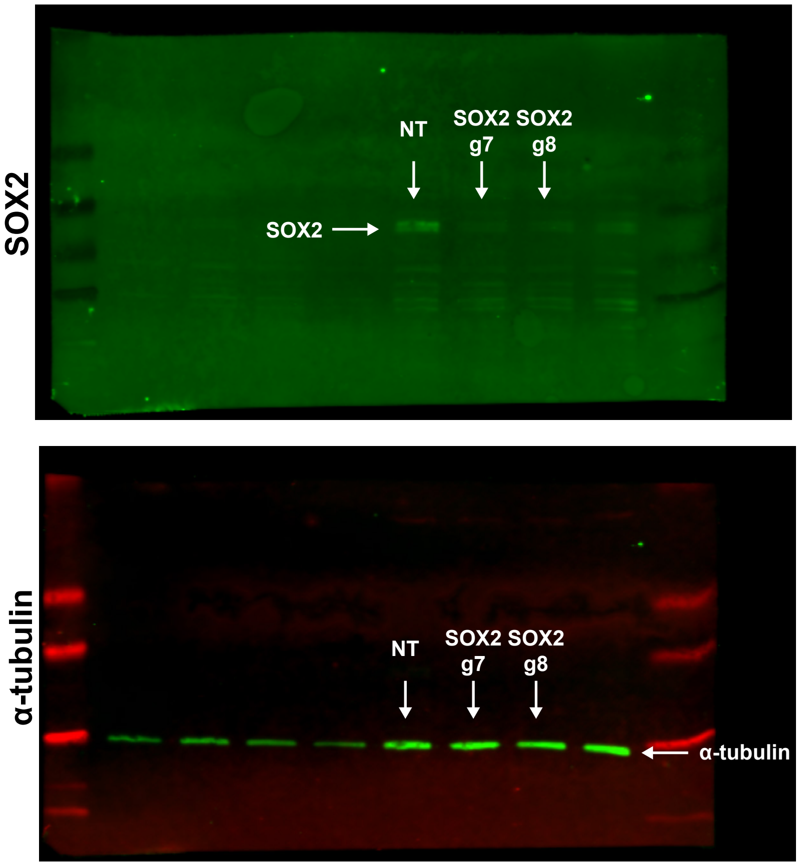

Figure 2-S2F

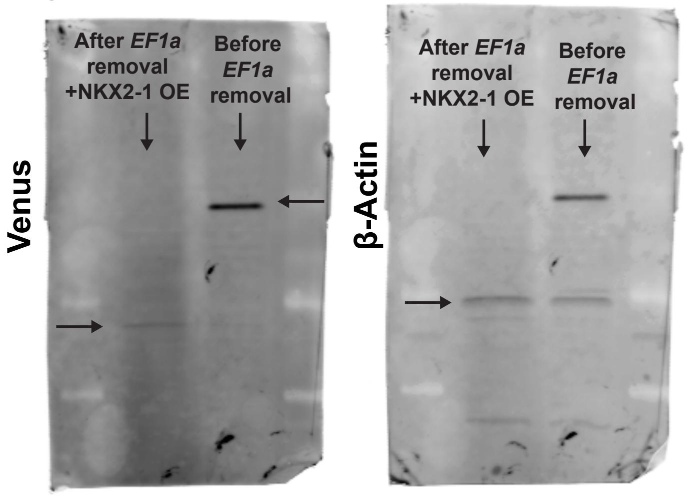

Please use ImageJ to open the raw data .TIF files.

Please use ImageJ to open the raw data .scn files

Figure 1-Figure Supplement 1c.

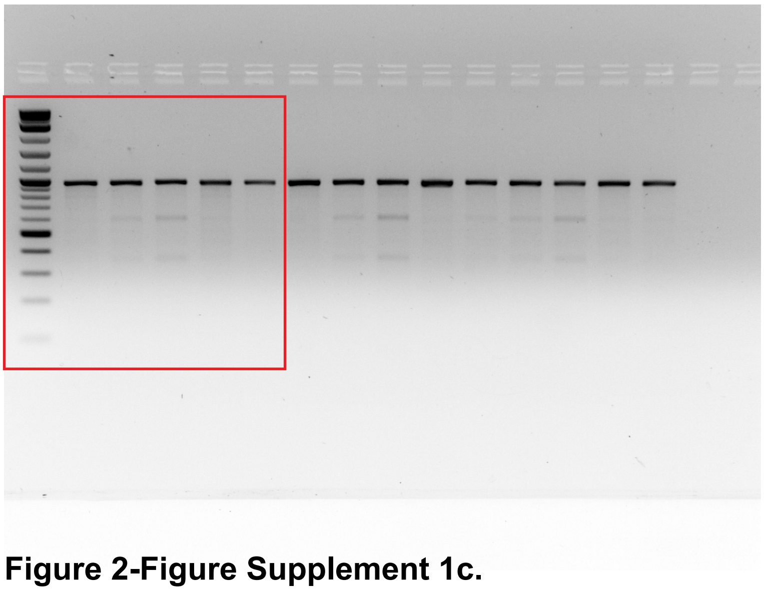

Red square labels the part of figure used

Figure 2-Figure Supplement 1c.

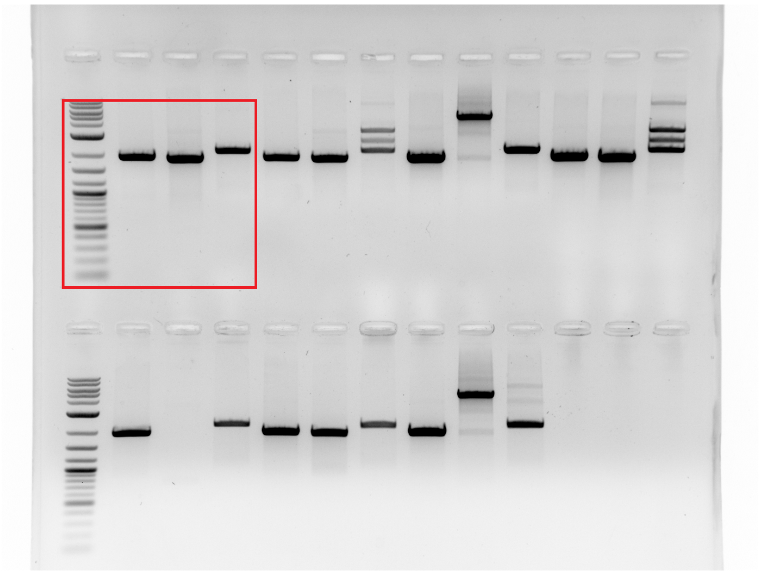

Red square labels the part of figure used

Figure 2-Figure Supplement 3b.

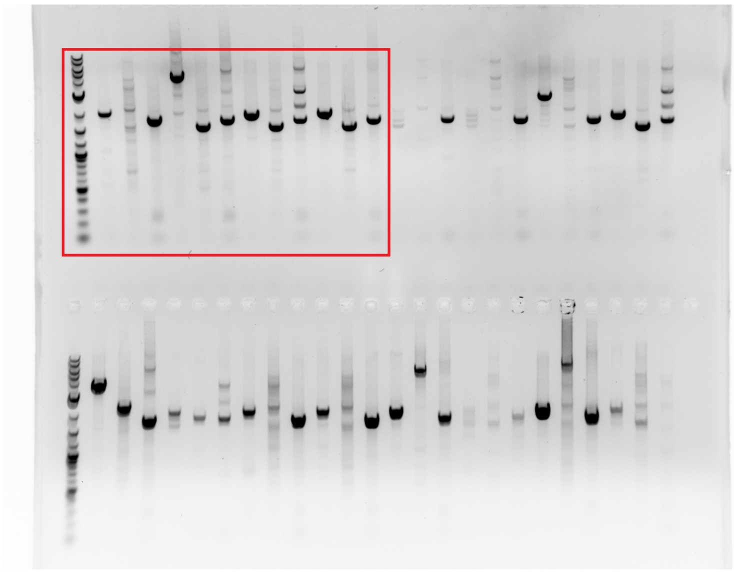

Red rectangle labels the part of figure used

Please use ImageJ to open the raw data .scn files

Figure 2-Figure Supplement 3e.

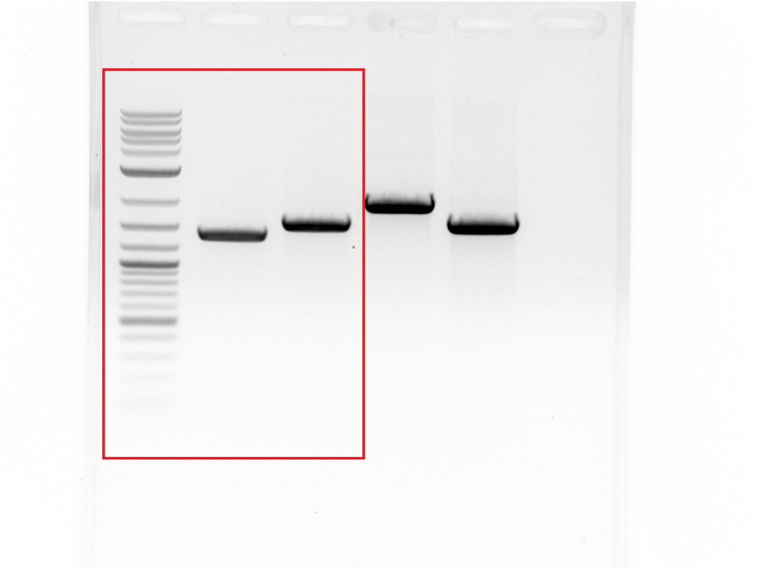

Red rectangle labels the part of figure used

Figure 2-Figure Supplement 4d.

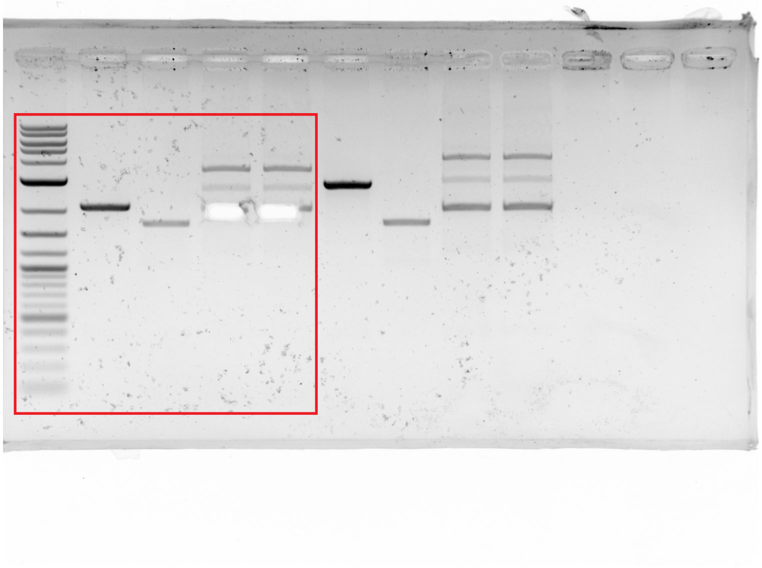

Red square labels the part of figure used

Figure 2-Figure Supplement 4f.

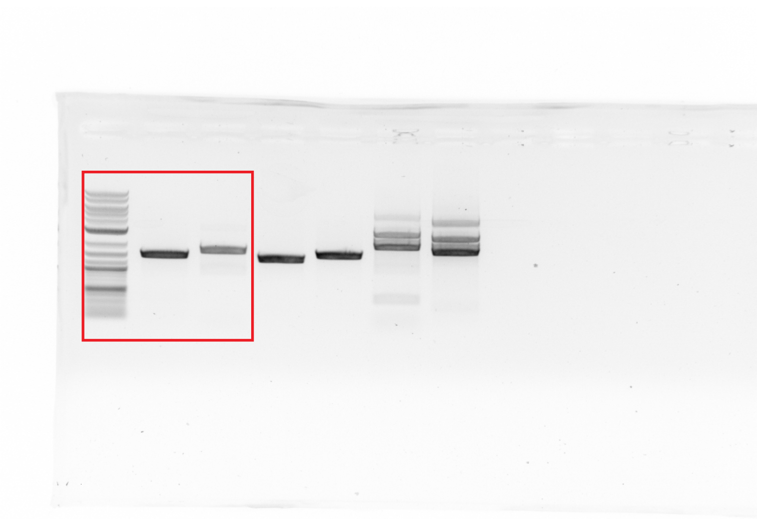

Red square labels the part of figure used

Please use ImageJ to open the raw data .scn files

Figure 2-Figure Supplement 5b.

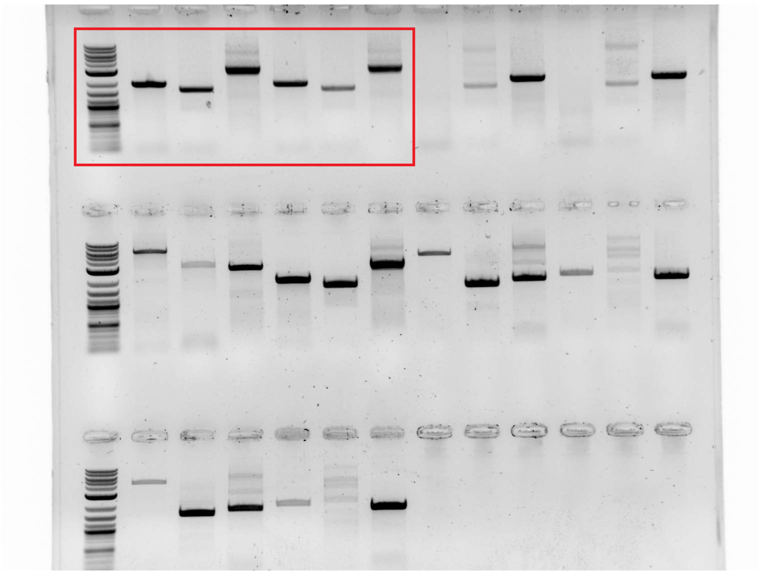

Red rectangle labels the part of figure used

Figure 2-Figure Supplement 5e.

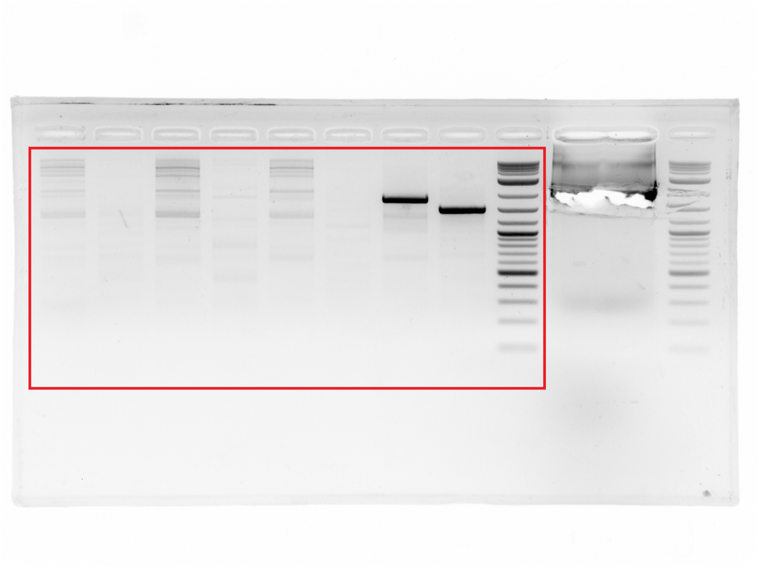

Red rectangle labels the part of figure used
